# Editorial: Association of metabolic diseases with cognition impairment and dementia

**DOI:** 10.3389/fendo.2023.1166342

**Published:** 2023-03-10

**Authors:** Sisi Luan, Jianbo Zhou

**Affiliations:** Department of Endocrinology, Beijing Tongren Hospital, Capital Medical University, Beijing, China

**Keywords:** metabolic diseases, cognition impairment, dementia, anti-diabetic agents, retinal imaging techniques

The current issue of Research Topic focuses on the impact of metabolic diseases on cognitive dysfunction, which ranges from mild cognitive impairment to dementia, and a total of 8 articles were included (4 original research, 3 reviews and 1 systematic review). The articles cover a variety of topics, including the effect of new hypoglycemic agents on cognition impairment and dementia, new methods for screening cognitive impairment and monitoring cognitive function, potential biomarkers identifying cognitive impairment, and the relationship of cognitive domain impairment with all-cause mortality.

Glucagon-like peptide 1 receptor (GLP-1R) agonists stand out among new diabetes drugs for their multiple neuroprotective mechanisms in Alzheimer’s disease (AD) models (**Figure 1** Du et al.) and their beneficial effect on a subset of type 2 diabetic patients (Luan et al.). In addition to GLP-1R agonists, another hypoglycemic drug, empagliflozin, can protect cognitive functions in obese mice. Empagliflozin may exert the above effect by inducing serine phosphorylation of MYH10, PAK4, and PIKfyve (Chen et al.). These recent discoveries reveal the potential of some new anti-diabetic agents as novel treatments for cognitive impairment and dementia.

**Figure 1:**
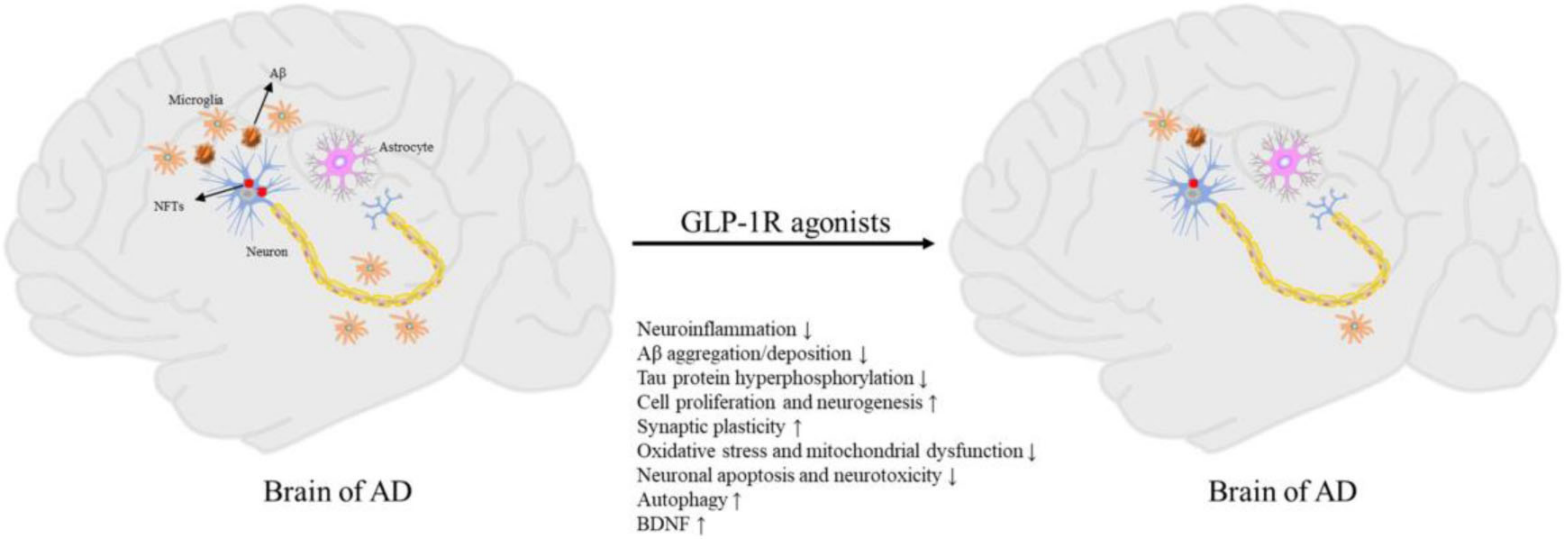
Pathogenesis of AD and neuroprotective effects of GLP-1R agonists (Du et al.).

In terms of new methods, due to the drawbacks of traditional imaging techniques, population-based data are lacking, and the association between the neurovascular coupling unit and the early stage of cognitive dysfunction remains unknown. It is worth noting that retinal imaging techniques are noninvasive, highly accurate, relatively inexpensive, and require a short measurement time. Neuronal and microvascular structures, as well as the function of the neurovascular coupling unit, as measured by retinal imaging, are linked to cognitive performance, suggesting a potential opportunity to collect population-based data to study the early pathobiology of cognitive dysfunction (van der Heide et al.). Similarly, while it is recommended that patients with diabetes over the age of 65 have an annual evaluation of cognitive function, the complexity and time-consuming nature of neuropsychological tests make routine incorporation into daily clinical practice unfeasible. Retinal microperimetry has emerged as a new reliable tool for the cognitive evaluation in patients with type 2 diabetes older than 65 years and may have the potential to change the current guidelines if preliminary results are confirmed in a large ongoing European trial (RECOGNISED) (Ciudin and Simo).

Due to the limitations of traditional evaluation methods, the identification of novel biomarkers for cognitive impairment and its related complications has been an active field of research. Data from a prospective cohort conducted by Tanaka et al. show that higher serum sTREM2 levels are a predictive marker for cognitive impairment in poorly controlled type 2 diabetes without obesity. NHANES cross-sectional study data show of a link between depression and blood urea nitrogen (Mao et al.). Furthermore, Guo et al. disclosed that executive and memory impairments may aid in the early identification of hemodialysis patients at risk of death. The preceding findings broaden our understanding of biomarkers of neuropsychological disease and emphasize the significance of assessing cognitive function.

As discussed, the Research Topic delves into various aspects of the links between metabolic diseases, cognition impairment, and dementia in both basic and clinical studies. We gratefully acknowledge all of the authors’ contributions and hope that the Research Topic will help advance the fields of endocrinology and neurology.

## Author contributions

SL and JZ contributed to the conception. SL drafted and revised the manuscript. All authors contributed to the article and approved the submitted version.

